# Comparison of the VersaTREK blood culture system against the Bactec9240 system in patients with suspected bloodstream infections

**DOI:** 10.1186/1476-0711-10-4

**Published:** 2011-02-05

**Authors:** Andries W Dreyer, Nazir A Ismail, Deliwe Nkosi, Kathy Lindeque, Marliza Matthews, Danie G van Zyl, Anwar A Hoosen

**Affiliations:** 1 Department of Medical Microbiology, University of Pretoria and Tshwane Academic Division of the National Health Laboratory Service (NHLS), Pretoria, South Africa; 2 Separation Scientific SA (Pty) Ltd, Johannesburg, South Africa; 3 Department of Internal Medicine, University of Pretoria, South Africa

## Abstract

**Background:**

To evaluate the VersaTREK (TREK Diagnostic Systems, Cleveland, Ohio) blood culture system against the Bactec9240 (BD Microbiology, Cockeysville, MD), for the recovery of bloodstream pathogens.

**Methods:**

Venous blood from patients with suspected bacterial sepsis was evenly distributed into bottles of each system. Positive signals were recorded and bottles processed onto standard media for organism recovery. False positive signals were regarded if no organisms were seen on Gram stain and no growth was observed.

**Results:**

177 bottles were available for analysis; the Bactec9240 system yielded 43 positive, 134 negative results and no false positive signals. The VersaTREK system had 58 positive signals with 14 being false positives.

**Conclusions:**

In our setting with high background burden of immuno-compromised patients, the VersaTREK system compared favourably with the Bactec9240 in recovering blood stream aerobic and facultative anaerobic pathogens from patients with suspected bacterial sepsis. A concern is the high false positivity rate. Due to its versatility to accommodate small and large workloads as well as using smaller volumes of blood, this system may establish itself as a useful alternative for the recovery of bloodstream pathogens.

## Background

The detection of bacteraemia is crucial for early and appropriate antimicrobial therapy. Blood cultures are considered as one of the most important specimen types and diagnostic laboratories process these as rapidly as possible. Despite newer molecular techniques being applied in diagnostic microbiology, recent analyses confirm the use of automated blood culture systems as the primary choice for detection of pathogens from blood specimens [1,2]. This is because results are generated rapidly compared to manual blood culture systems and antimicrobial susceptibility testing can also be performed which is a limitation of molecular techniques.

In South Africa, with its human immune-deficiency virus (HIV) burden, patients are at an increased risk of developing sepsis due to bacterial, fungal and mycobacterial infections [3]. Most established referral diagnostic laboratories in South Africa use large automated blood culture systems. However, there is a need for smaller blood culture systems to be placed in laboratories attached to regional hospitals which may serve rural populations. The various blood culture systems compete for higher sensitivity for recovery of blood-borne pathogens as well as a faster time to detection (TTD). A number of studies have shown the use of TTD as a good predictor of clinical outcome in staphylococcal sepsis [4-6].

The most frequently used systems in South Africa are the BacT/Alert^® ^3D System (bioMérieux, Durham, N.C.) and the Bactec9240™ System (BD Microbiology, Cockeysville, MD). The VersaTREK^® ^System (Trek Diagnostic Systems, Cleveland, Ohio) has recently entered the market in South Africa. The critical choice of which automated blood culture system to use in a laboratory is influenced by a number of factors. These include TTD, sensitivity for organism recovery, workload capacity, user interface and costs. The automated blood culture systems differ to some extent in the method used to detect microbial growth. The Microbiology Diagnostic Laboratory at our academic complex serves a large tertiary hospital as well as two regional hospitals and uses the Bactec9240 system with a workload capacity of 240 bottles.

The VersaTREK system can be adapted to accommodate small or big volumes of blood culture bottles and with its 96 bottle capacity is ideal for smaller laboratories. This system also has the advantage of using small volumes of blood from as little as 0.1 mL to 10 mL. The 80 mL broth creates an optimal blood-to-broth ratio of 1:9 to minimize, by dilution, the effect of host serum factors and antimicrobials. Aerobic bottles are vortexed with a stir bar to enhance oxygenation of the broth. The media is suitable for all patient populations. The VersaTREK system is based on monitoring pressure changes in the bottle headspace due to bacterial metabolism and these changes are measured every 24 minutes according to the VersaTREK instrument specifications. This system is neither limited by organisms that produce low CO_2 _concentrations as it detects any gas produced or consumed by microorganisms nor by a high amount of white blood cells.

There has been only one published study which compared the VersaTREK system to the BacT/Alert system using clinical isolates [7]. Our study is the first to compare the VersaTREK system to the Bactec9240 system using clinical samples from patients with suspected bacterial sepsis in the high HIV prevalence setting of South Africa.

## Methods

The study was conducted in the National Health Laboratory Service (NHLS), Tshwane Academic Division, Diagnostic Microbiology Laboratory at the University of Pretoria, South Africa.

Venous blood was collected via a sterile syringe from 74 adult patients at a provincial hospital, suspected of having a blood stream infection. The patients were mainly from the adult medical wards and the intensive care unit. This study however has no clinical data as it is a direct laboratory based comparison between two systems. Skin disinfection was performed prior to collection with 70% alcohol and allowed to air dry. Clinicians were instructed to inoculate a volume of 5 mL venous blood into the respective bottles for each system. For the VersaTREK system this included VersaTREK REDOX 1^® ^(aerobic) (TREK Diagnostic Systems, Cleveland, Ohio) and VersaTREK REDOX 2^® ^(anaerobic) bottles and for the Bactec9240 system Bactec™ Plus Aerobic/F and Bactec™ Plus Anaerobic/F bottles (Becton Dickinson, Sparks, MD). All bottles were received within normal working hours and incubated in their respective systems within two hours of receipt.

Once a signal was detected on any system the TTD was recorded. This was followed by performing Gram staining and inoculating two non-selective agar plates, a blood agar and a chocolate agar plate, as well as a selective and differential plate, MacConkey agar, for each signal positive bottle. These were incubated aerobically at 35°C for 18 - 24 hours and observed for growth. Anaerobic bottles received an additional *Brucella *blood agar plate that was incubated anaerobically for 7 days. The Vitek 2™ system (bioMérieux, Durham, USA) was used for organism identification and susceptibility testing by using the Vitek 2 GN and AST-N133 cards respectively. A false positive signal was defined as a true signal emitted from the instrument, but no organisms were seen on the Gram stain and no growth was obtained after 48 hrs extended incubation. False positive signals were further evaluated by examining the instrument growth curves for exponential bacterial growth and documenting the time to removal (TTR) of the bottles. Blood culture bottles were regarded as negative after 7 days incubation with no positive signal being emitted.

Only isolates with a printed record of the exact TTD on the Bactec system as well as a growth curve with TTD and TTR on the VersaTREK system was included for comparison. For statistical analysis, the Paired *t*-Test was used to calculate the mean for TDD of isolates in which TDD was recorded. A p-value of less than 0.05 was considered statistically significant. Data was analysed using Microsoft Office Excel 2003 software. The TTD was averaged for all isolates with comparable culture results and further stratified according to gram-positive and gram-negative organisms. Positive and negative percent agreements between the two systems were determined for both organism recovery and TTD [8].

## Results

A total of 177 comparative bottles were analysed in the laboratory. The Bactec9240 system yielded 43(24%) positive signals and no false positive signals. The VersaTREK system had 58(33%) positive signals of which 44(76%) were true positive and 14 (24%) were false positive signals (Table 1). There were 5 discrepant results. Of the 60 positive signals detected, 31(52%) were from aerobic bottles and 29(48%) from anaerobic bottles. The culture positivity rate for the VersaTREK and the Bactec9240 excluding false positive results was similar i.e. 25% (44 of 177) and 24% (43 of 177) respectively. The most productive bottle in both systems was a VersaTREK Redox 1 bottle that had a positive signal at 1 hour and 6 minutes and isolated *Staphylococcus aureus *with the corresponding Bactec bottle flagging at 3 hours and 3 minutes.

**Table 1:** Results for bottles which gave positive signals

	Total positive signals	Positive on both systems	Positive on one system	False positive signals
Bactec9240 (N = 177)	43	41	2	0
VersaTREK (N = 177)	58	41	3	14

The results were further analysed by organisms isolated in each system (Table 2). A total of 50 organisms were isolated out of 46 bottles (i.e. 41 positive on both systems plus the 5 positive by any one system). VersaTREK detected an additional three isolates which included viridans streptococcus, *Enterococcus faecalis *and *Bacillus *species. The Bactec9240 system detected two additional isolates which were both *Streptococcus pneumoniae*.

**Table 2:** Total organisms recovered by both systems (N = 50)

	VersaTREK + Bactec9240 +	Only VersaTREK +	Only Bactec9240 +
**Gram-positives**			
*Streptococcus pneumoniae*	**8**	**0**	**2**
Viridans streptococcus	**1**	**1**	**0**
*Enterococcus faecalis*	**2**	**1**	**0**
*Staphylococcus aureus*	**10**	**0**	**0**
Coagulase negative staphylococci	**4**	**0**	**0**
*Bacillus *species	**0**	**1**	**0**
**Gram-negatives**			
*Klebsiella pneumoniae*	**5**	**0**	**0**
*Proteus mirabilis*	**2**	**0**	**0**
*Pseudomonas aeruginosa*	**2**	**0**	**0**
*Acinetobacter baumannii*	**2**	**0**	**0**
*Stenotrophomonas maltophilia*	**1**	**0**	**0**
*Serratia marcescens*	**4**	**0**	**0**
*E. coli*	**2**	**0**	**0**
*Enterobacter *species	**2**	**0**	**0**
**Total**	45* (41bottles)	**3**	**2**

* = A total of 41 positive bottles with 4 bottles yielding two organisms each

For the two discrepant *Streptococcus pneumoniae *isolates, the VersaTREK system emitted positive signals but no organisms were seen on Gram stain nor was any growth observed after 48 hours incubation. The VersaTREK system also emitted an additional 12 positive signals which were Gram stain smear negative and yielded no growth and were therefore also considered to be false positives. The instrument growth curves of the pressure changes plotted against time for these bottles were evaluated and did not indicate logarithmic bacterial growth. The average TTR for these bottles was 8 hours.

The TTD for the two systems is shown in Table 3. Thirty-one bottles had matching TTD data available for comparative analysis. No statistically significant difference in TTD was observed between the two systems. The positive and negative analytical percent agreements for organism recovery were 95% and 98% respectively. The two systems were also compared for signal detection. The positive and negative analytical percent agreements for signal detection were 95% and 87% respectively.

**Table 3:** Time to detection (TTD) in hours (N = 31)

	VersaTREK	Bactec9240	Mean difference	95% CI	P value
Average for all isolates (N = 31)	20.41	16.86	3.55	-5.8583 - 12.9551	0.45
Average for GP N = (17)	10.28	8.35	1.92	-4.0052 - 7.8570	0.50
Average for GN (N = 14)	32.71	27.20	5.52	-15.6667 - 26.7038	0.58

GP = Gram positives

GN = Gram negatives

## Discussion

This is the first study to report a comparison of the VersaTREK system to the Bactec9240 system using clinical samples from patients with suspected bloodstream infections. The study was carried out in South Africa where HIV infection rates are very high and hence a greater risk exists among immune compromised patients to develop bloodstream infections [3,9]. The true culture positivity rate for both blood culture systems was high (25%). Although this is much higher than what is usually reported (range 10 - 15%) from other centres [7,10,11] it does not come as a surprise considering the high HIV endemicity in the country, the intensive care setting and the struggle to adhere to good infection control practices.

Reports of comparisons of blood culture systems have appeared in the literature but these have been mainly between the various Bactec systems and the BacT/Alert system [10,11]. There has been only one published study which evaluated the VersaTREK blood culture system and this comparison was against the BacT/Alert system [7]. Overall we detected a fairly good correlation between the two systems that we evaluated. However, the false positive signals emitted from the VersaTREK system accounted for a false positivity rate of 7.9%. This is much higher than that reported in Mirrets' study [7].

Various reasons for the false positives must be considered. A delay in processing the bottles post signal detection may result in negative growth of fastidious organisms as they may be susceptible to autolysins as was noted with the two *Streptococcus pneumonia *isolates. Post incubation processing was delayed in some cases as the laboratory was not continuously staffed and this may be a factor as the average TTR for the false positive signal bottles was 8 hours. Other organisms may require additional culture media e.g. nutritionally variant streptococcus and *Campylobacter spp*. Non-cultivable organisms may only be identified by molecular techniques. Prior antibiotic use could be a factor even though the media does contain antibiotic binding resin. The issue of high white cell counts could not be addressed in this study, although this system is stated not to be limited by this factor. Another possibility for the false positive signals may be that the threshold setting of the system for detection is too low. Furthermore, the system also vortexes the aerobic bottles compared to the slow rocking method employed by the Bactec9240 system and this may also contribute to additional false signals by creating pressure changes. However, there is no evidence to support our hypotheses.

A change in the spectrum of organisms being isolated from episodes of sepsis has been noted over the last few years [3,9]. The increased use of more invasive therapeutic devices as well as the administration of immunosuppressant agents has increased the incidence of septicaemia. Studies have also shown a decline in the recovery of *Streptococcus pneumoniae *and other opportunistic pathogens with the introduction of the pneumococcal conjugated vaccine and highly active antiretroviral therapy - HAART [3,9].

The organisms recovered in our study represent a good variety of potential pathogens that can cause a wide spectrum of clinical symptoms and disease. The Bactec9240 detected two additional isolates of *Streptococcus pneumoniae*. It must be noted that these isolates did emit a positive signal on the VersaTREK system, at 8 and 10 hours respectively, however these bottles were removed and processed after 24 hours owing to the closure of the blood culture laboratory overnight. Owing to the sensitive nature of this organism it might explain the poor recovery rate in this specific instance. The VersaTREK detected additional organisms such as viridans streptococcus and *Bacillus *species which are often contaminants. In this study all these patients had normal inflammatory markers suggesting probable contamination (data not shown). Overall if one considers clinically significant blood culture isolates, this study demonstrated a fairly good analytical percent agreement for organism recovery.

Our analysis regarding the time to detection revealed a faster overall TTD for all isolates with the Bactec9240 system although this was not statistically significant. Variations in the propriety culture media in each blood culture bottle including their additional nutrients and anti-coagulants may be contributory factors. Furthermore, the Bactec9240 systems' detection of CO_2 _release may be faster than the VersaTREK system that measures headspace pressure. Although there was no statistical significance in the TTD the difference may have clinical bearing when treating gram-negative sepsis.

We cannot comment on the efficiency of this system for the recovery of fungi and anaerobes as these were not recovered during the study period. However, in another study the VersaTREK system was comparable to the BacT/Alert for the recovery of yeasts from blood cultures [7].

Our study was limited by the fact that we could not assess the false positive signals by a lack of knowledge of prior antimicrobial usage or the means to detect bacterial 16S rRNA or fungal genomic sequences. Another limitation was the relatively small number of positive isolates for the calculation of the TTD for the two systems, however performance data in a real world setting is often difficult to obtain and always useful. Although this study was mainly laboratory based, additional clinical data and information regarding outcomes could have added additional weight to our conclusions. The issue of false negative cultures could not be addressed. Another approach could have been to perform terminal cultures from the negative bottles in those isolates that were missed by the Bactec9240 system as well as the false positive signals, however this was not part of the study design. The ability of the VersaTREK system to work with much lower volumes was not assessed in this study as we used equal volumes to compare both systems, this however may be a major advantage of the system especially in the paediatric population. We made use of one clinician to oversee the blood culture collection process, however this was not evaluated and the likely limited variation might have bearing on the false positive rate.

## Conclusions

In our setting with high background burden of immuno-compromised patients due to HIV as well as a higher prevalence of blood stream infections, the VersaTREK system compared favourably with the Bactec9240 in recovering blood stream pathogens from patients with suspected bacterial sepsis. However, a concern is the high false positivity rate observed in our study. Due to its versatility to accommodate small and large workloads as well as the added benefit of using much smaller volumes of blood, this system may establish itself as a useful alternative for the recovery of bloodstream pathogens.

## Competing interests

The authors declare that they have no competing interests.

## Authors' contributions

AWD was involved in data analysis, drafting and submission of the manuscript. NAI was involved in drafting and critical review of the manuscript. DN was involved in the statistical analysis of results. KL was overseeing and coordinating the laboratory component. MM was responsible for data analysis and technical support. DGZ co-ordinated the distribution, collection and transport of the blood culture bottles at the provincial hospital. AAH was involved in drafting and critical review of the manuscript. All the authors approved the final manuscript.
